# Extracellular vesicles miR‐210 as a potential biomarker for diagnosis and survival prediction of oral squamous cell carcinoma patients

**DOI:** 10.1111/jop.13263

**Published:** 2021-12-01

**Authors:** Elisabetta Bigagli, Luca Giovanni Locatello, Arianna Di Stadio, Giandomenico Maggiore, Francesca Valdarnini, Franco Bambi, Oreste Gallo, Cristina Luceri

**Affiliations:** ^1^ Department of Neuroscience, Psychology, Drug Research and Child Health – NEUROFARBA – Section University of Florence Florence Italy; ^2^ Department of Otorhinolaryngology Careggi University Hospital Florence Italy; ^3^ Department of Otolaryngology Silvestrini University Hospital University of Perugia Perugia Italy; ^4^ Cell Factory Meyer "A. Meyer" University Children's Hospital Florence Italy

**Keywords:** biomarkers, diagnosis and prognosis, extracellular vesicles, miRNA, oral cancer

## Abstract

**Background:**

The identification of early diagnostic and prognostic biomarkers in oral squamous cell carcinoma (OSCC) is an unmet clinical need. We hypothesized that extracellular vesicles miR‐210 expression (EV‐miR‐210) could be a potential biomarker for OSCC diagnosis and follow‐up.

**Methods:**

The expression of EV‐miR‐210 was measured in the plasma of OSCC patients (*n* = 30) and compared to that of controls (*n* = 14).

**Results:**

The median EV‐miR‐210 expression was significantly higher in OSCC patients compared to controls who had often, undetectable levels (*p *< 0.0001). We performed receiver operating characteristic (ROC) analysis for discriminating OSCC cases from controls. EV‐miR‐210 yielded an area under the curve (AUC) of 0.9513 with sensitivity 92.3% and specificity 86.6%. Kaplan‐Meier curves indicated that high EV‐miR‐210 expression was associated with worse 3 years’ survival (*p *< 0.05). Cox regression hazard model indicated that high EV‐miR‐210, G2, and G3 grading and pathological nodal status (pN)>1 were independent predictors of worse survival in OSCC patients.

**Conclusion:**

These preliminary data suggest that EV‐mir‐210 may be a novel diagnostic and prognostic biomarker in OSCC.

## 1 INTRODUCTION

Oral cancer, predominantly in the form of oral squamous cell carcinoma (OSCC), is a major cause of the worldwide morbidity and mortality.^1^ Despite important improvements in surgical and non‐surgical techniques, OSCC still presents a recurrence rate of 25%–45%^2^, ^3^ without significant variation of the survival rate at 5 years over the last thirty years.^4^ In addition, more than ⅔ of OSCC cases are still diagnosed at advanced stages, and such a delay has an enormous impact on OSCC‐related mortality and costs.^5^

Early detection of OSCC could allow treatment of OSCC in the early phases, positively affecting the survival rate. Unfortunately, the current available methods are invasive and discomforting especially in case of multiple solid biopsies. In this context, liquid biopsy, based on the detection of tumor cells, nucleic acids, and extracellular vesicles (EVs) in body fluids has emerged as a revolutionary, non‐invasive tool for the early diagnosing and prognosis, minimal residual disease detection, and prediction of treatment response in oral cancer.^6^ EVs are small, membrane‐coated particles containing proteins, lipids, DNA, mRNAs, and microRNAs (miRNAs). EV‐miRNAs play an important role in the intercellular communication between the tumor and the microenvironment and are involved in oral cancer development and progression..^7^

MiR‐210 is the most responsive, influential miRNA regulated under hypoxic conditions and it is involved in several key biological processes including proliferation, apoptosis, DNA repair, metabolism, angiogenesis, and immune responses.^8^

We reported that miR‐210‐3p was significantly upregulated in exosomes, a subtype of EVs secreted by colon cancer cells and correlated to anoikis resistance and epithelial‐mesenchymal markers.^9^ Jung et al. found that exosomes containing miR‐210 are transferred in the tumor microenvironment, regulate the expression of vascular remodeling genes and promote angiogenesis in breast cancer cells.^10^ Similarly, the delivery of exosomal miR‐210‐3p in human umbilical vein endothelial cells promoted oral cancer angiogenesis through the PI3K/AKT signaling pathway.^11^ Lu et al. also reported that hypoxia‐driven miR‐210‐3p expression enhanced oral cancer cell proliferation and clone formation.^12^

Currently, there is no evidence of the use of miR‐210 as a potential biomarker for OSCC. The aim of the present study was to evaluate whether circulating extracellular vesicles from OSCC patients and healthy controls express different levels of miR‐210 and whether it may be applied as a potential non‐invasive biomarker for OSCC diagnosis and follow‐up.

## 2 PATIENTS AND METHODS

### 2.1 Clinical specimens

A total of 30 OSCC patients undergoing surgery were enrolled at the Department of Otorhinolaryngology, Careggi University Hospital, Florence. This study was conducted according to the criteria set by the Declaration of Helsinki. The study was approved by the local research ethics committee (EC code: OSS_10/2010), and informed consents were obtained from all participants. Venous blood (5 ml) from each patient was collected at surgery. The whole blood was separated into plasma by centrifugation at 1200×*g* for 10 min. The plasma was stored at −80°C until analysis. Moreover, 14 plasma samples were obtained from healthy blood donors enrolled at the Transfusion Unit of the Meyer Hospital, Florence, Italy. The median follow‐up of OSCC patients was 110 months (range: 6–123 months). Information on smoking habit and alcohol consumption were obtained from patient's medical records (Table 1).

**TABLE 1:** Characteristics of OSCC patients and healthy blood donors

Characteristics	Healthy blood donors *n *= *14*	OSCC patients *n *= *30*	*p* value
Age (years)	51.5 (46–59)	65.0 (61–79)	<0.001
Gender (*n*, %)			>0.999
Female	4 (28.6%)	8 (26.7%)	
Male	10 (71.4%)	22 (73.3%)	
Smoke habit (*n*, %)			<0.0001
No smokers	14 (100%)	9 (30%)	
Smokers	0	21 (70%)	
Alcohol abuse (*n*, %)			0.1607
Yes	0	5 (16.7%)	
No	14 (100%)	25 (83.3%)	

Note

Data are expressed as median (interquartile range, IQR) or number (percentage)

### 2.2 Isolation of extracellular vesicles from plasma samples

Extracellular vesicles were isolated from plasma by using the Exosome Precipitation Solution (Machery‐Nagel), following manufacturer's instructions. Briefly, Exosome Precipitation Solution (400 μl) was added to 1 ml of plasma, and samples were vortexed for 10 s, incubated for 30 min at 4°C and then centrifuge for 5 min at >500 × *g* to collect the pellets.

### 2.3 Extraction of total RNA

Total RNA was extracted using the miRNeasy Serum/Plasma Kit (Qiagen) according to the manufacturer's instructions. Briefly, 500 µl of QIAzol Lysis Reagent (Qiagen) was added to extracellular vesicles pellets, mixed and incubated at room temperature for 5 min. Following the addition of chloroform, through mixing and centrifugation to separate organic and aqueous phases, the aqueous phase was recovered and mixed with ethanol. The sample ethanol mixture was added to an RNeasy MinElute spin column and centrifuged. The column was washed once with buffer RWT, and then twice with buffer RPE followed by elution of RNA in 14 µl water.

### 2.4 Quantification of miRNA by qPCR

Total RNA (100 ng) was retro‐transcribed to cDNA with the miScript II RT Kit (Qiagen, Hilden, Germany) In brief, 1.5 μl total RNA was mixed with 2 μl 5× miScript HiSpect Buffer, 1 μl 10× miScript nucleics mix, and 1 μl miScript Reverse Transcriptase mix and made up to 10 μl with H_2_O. Reverse transcription was performed under the following conditions: 37°C for 60 min and 95°C for 5 min. The resulting cDNA was diluted by adding 40 µl RNase‐free water and pre‐amplified by using the miScript PreAMP PCR Kit (Qiagen). In brief, 5 µl of the diluted cDNA was mixed with 5 µl 5× miScript PreAMP buffer, 2 µl HotStar Taq DNA polymerase, 1 µl miScript PreAMP universal primer, 5 µl miScript PreAMP primer mix and final volume 25 µl made up with RNase‐free water. Pre‐amplification of cDNA was carried out as follows: 95°C for 15 min followed by 12 cycles with denaturation at 94°C for 30 s and annealing at 60°C for 3 min. After pre‐amplification, the cDNA was diluted 5‐fold with nuclease‐free water and stored in −80°C until use. qPCR was carried out in a 7900 Real‐Time PCR System (Applied Biosystems) using the miScript^®^ SYBR^®^ Green PCR Kit (Qiagen). qPCR was performed in triplicate using 20× QuantiTect syber green PCR master mix and 10× miScript universal primer. The data were analyzed with automatic setting for assigning baseline; the threshold cycle (Ct) was defined as the cycle number at which the fluorescence exceeded that of the given threshold. The presence of a single sharp peak in the melt curve at the end of the PCR cycles confirmed the specificity of primer annealing.

As there is no consensus on endogenous miRNA that can reliably serve as a “reference gene” that is stably detected in extracellular vescicles, the levels of miR‐210‐3p were normalized against miR‐16‐5p, previously used as a reference gene for normalizing miR‐210‐3p expression^13^ and showed no changes in our OSCC patients compared to controls, in agreement with the results of Momen‐Heravi et al.^14^

ΔCT was calculated by subtracting the CT values of mir‐16 from the CT values of miR‐210‐3p. ΔΔCT was then determined by subtracting the average ΔCT of controls from the ΔCT of patients. The relative expression levels of miR‐210 were determined using the following equation: 2^−∆∆CT^. The 30 OSCC patients were categorized into high and low ev‐miR‐210 expression groups (15 patients each), according to their median expression value (19.70) as the threshold.

### 2.5 Mir‐210‐3p expression in tumor tissue from head and neck squamous cell carcinoma (HNSCC) patients according to data from the cancer genome atlas

We used miRNA Target Viewer (miR‐TV),^15^ to interrogate the expression data of mir‐210‐3p in tumor and healthy tissues from in head and neck squamous cell carcinoma (HNSCC) patients. miR‐TV is a web tool, based on The Cancer Genome Atlas (TCGA) program,^16^ a well‐known large‐scale cancer research program for studies on human cancers.

### 2.6 Statistical analysis

Comparison of continuous variables between groups was performed using the Student's *t*‐test (normally distributed) or Mann‐Whitney test (non‐normally distributed). Differences between proportions were assessed using the Fisher exact test. To identify independent factors affecting EV‐miR‐210 levels, a multiple regression analysis was performed, including as variables, age, and smoke habit. The area under the curve (AUC) of a receiver operating characteristic (ROC) curve was used to estimate the ability of the measure of circulating EV‐miR‐210 to discriminate OSCC patients from healthy subjects. The optimal cut‐off value, sensitivity, and specificity were determined by calculating the maximum likelihood ratio (LR).

The overall survival was calculated stratified OSCC patients into two groups, according to the median level of circulating EV‐miR‐210, at 3 years and at the maximum follow‐up time available from the date of surgery. Survival curves were generated using the Kaplan‐Meier method and evaluated by log‐rank test. Multivariable survival analysis was performed using the Cox proportional hazard model with variables likely affecting the outcome (T, N, tumor differentiation, resection margin, smoking, and alcohol behavior) and the EV‐miR‐210 levels, in a backward selection model.

P‐values less than 0.05 were considered statistically significant. Statistical analyses were performed using SPSS statistics 27 software (IBM Corp.) and GraphPad Prism 7.0 (GraphPad Software).

## 3 RESULTS

### 3.1 Characteristics of OSCC patients and healthy volunteers

There were no significant differences in gender distribution or use of alcohol between OSCC patients and healthy controls. On the contrary, healthy volunteers were significantly younger and were all no smokers (Table 1). As shown in Figure S1, panel A and B, EV‐miR‐210 expression did not correlate with age in OSCC patients (*p *= 0.0.6535) and did not differ between smokers and non‐smokers (*p *= 0.5413); moreover, multivariate regression analysis performed on OSCC patients revealed that age and smoke habit were not associated with EV‐miR‐210 expression (Table S1).

### 3.2 Plasma miR‐210 expression is increased in EVs from OSCC patients

Given the rising interest in the functions of EVs, the International Society of Extracellular Vesicles gave some recommendations on their characterization.^17^ In this study, a commercial kit claiming “exosome” purification from plasma was used, but since no further characterization in terms of size of the particles isolated or specific marker expression was performed, our results are expressed in terms of EV‐miR‐210 expression instead of exosomal miR‐210.

**FIGURE 1:**
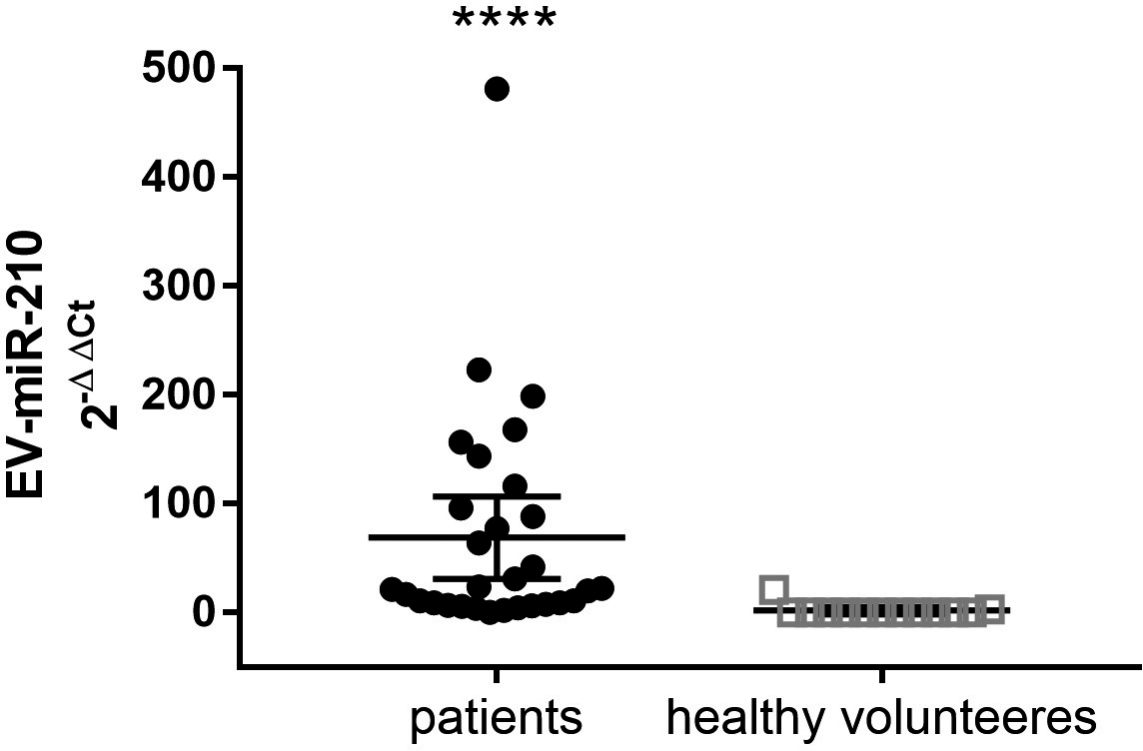
Expression levels of EV‐miR‐210 from plasma samples of OSCC patients (*N* = 30) and healthy volunteers (*N* = 14). Data are expressed as median with IQR **** *p *< 0.0001 by Mann‐Whitney test

As shown in Figure 1, EV‐miR‐210 levels were significantly increased in plasma samples of OSCC patients (median (interquartile range, IQR) 19.70 (7.1–101.2) compared with those of healthy blood donors whose levels were very low and, often, undetectable (0.1 (0.1–0.4)), *p *< 0.0001 by Mann‐Whitney test).

### 3.3 Ev‐miR‐210 as a diagnostic biomarker for OSCC patients

**FIGURE 2:**
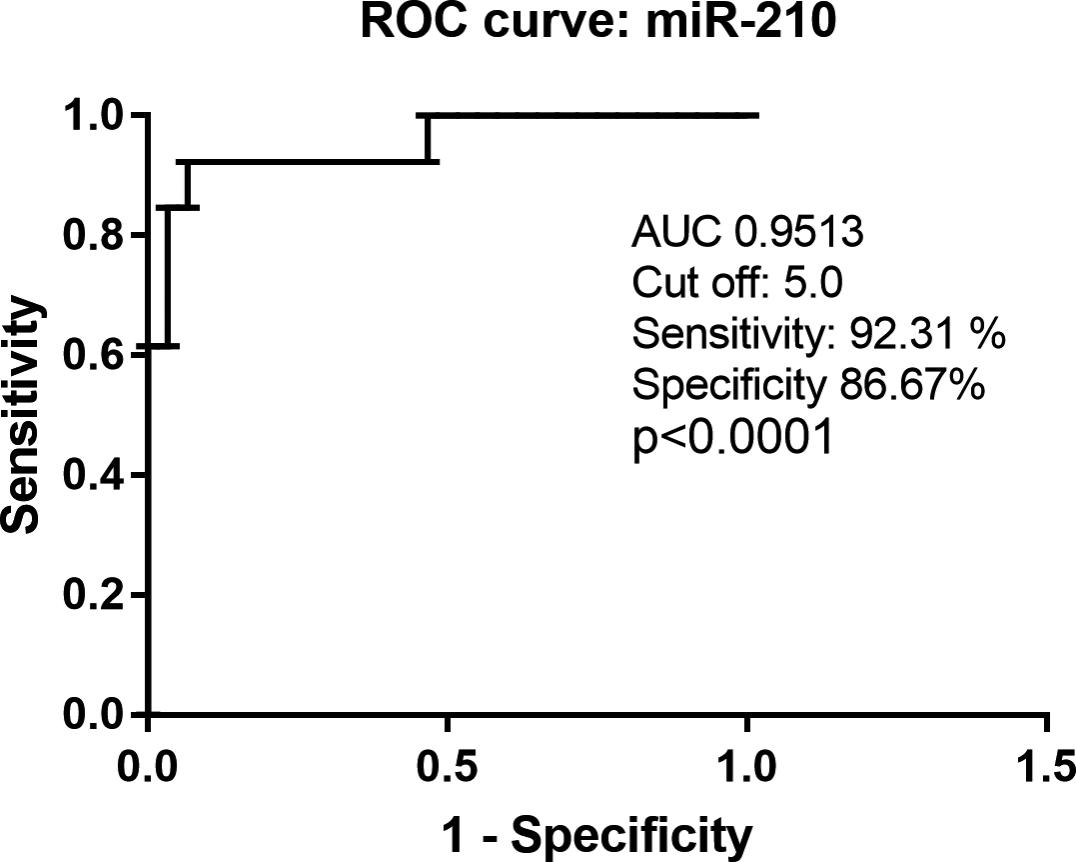
Receiver operating characteristic (ROC) curve analysis of EV‐miR‐210 expression levels. EV‐miR‐210 yielded an AUC of 0.9513 with a sensitivity of 92.31% and a specificity of 86.67% for discriminating OSCC patients from healthy controls (*p *< 0.0001)

Receiver operating characteristic curve analysis showed that EV‐miR‐210 had a high clinical value in the diagnosis of OSCC. As shown in Figure 2, in fact, EV‐miR‐210 levels performed very well in distinguishing patients from healthy controls, with an AUC value of 0.9513 (95% CI, 0.8777–1.025), *p *< 0.0001. The optimal threshold value of EV‐miR‐210 expression was set at <5 that corresponds to the maximum likelihood ratio (LR) of 6.92, with a sensitivity of 92.31% and a specificity of 86.67%.

### 3.4 Correlation between EV‐miR‐210 expression and clinicopathological features of OSCC patients

The median expression of EV‐miR‐210 divided OSCC patients into high and low expression groups, and the clinicopathological data of the two groups were compared (Table 2). No significant associations were observed, although we noted a higher pathological nodal status (pN) in patients from the high EV‐miR‐210 expression group (*p *= 0.1281).

**TABLE 2:** Characteristics of OSCC patients according to EV‐miR‐210 expression

Characteristics		<miR−210	>miR−210	*p* value
*n*	*n* = 30	*n* = 15	*n* = 15	
Age (years)	65.0 (61–79)	64.0 (60–74)	69.0 (64–80)	0.1285
Gender				0.6817
Female	8 (26.7%)	5 (33.3%)	3 (20.0%)	
Male	22 (73.3%)	10 (66.7%)	12 (80.0%)	
Smoke habit				>0.9999
No smokers	9 (30.0%)	4 (26.7%)	5 (33.3%)	
Smokers	21 (70.0%)	11 (73.3%)	10 (66.7%)	
Alcohol abuse				0.3295
Yes	5 (16.7%)	4 (26.7%)	1 (6.7%)	
No	25 (83.3%)	11 (73.3%)	14 (93.3%)	
Status				0.1281
Dead	11 (36.7%)	3 (20.0%)	8 (53.3%)	
Alive	18 (60.0%)	12 (80.0%)	7 (46.7%)	
T classification				0.5924
1	14 (46.7%)	6 (40.0%)	8 (53.3%)	
2	6 (20.0%)	3 (20.0%)	3 (20.0%)	
3	7 (23.3%)	5 (33.3%)	2 (13.3%)	
4	3 (10.0%)	1 (6.7%)	2 (13.3%)	
N classification				0.1281
0	19 (63.3%)	12 (80.0%)	7 (46.7%)	
>1	11 (36.7%)	3 (20.0%)	8 (53.3%)	
Grading				0.6817
G1	21 (70.0%)	11 (73.3%)	10 (66.7%)	
G2‐G3	9 (30.0%)	4 (26.7%)	5 (33.3%)	
Resection margin				0.4828
Negative	28 (93.3%)	13 (93.3%)	15 (100%)	
Positive	2 (6.7%)	2 (13.3%)	0	
Perineural invasion				>0.9999
Yes	10 (33.3%)	5 (33.3%)	5 (33.3%)	
No	20 (66.7%)	10 (66.7%)	10 (66.7%)	
Vascular invasion				0.3898
Yes	7 (23.3%)	5 (33.3%)	2 (13.3%)	
No	23 (76.7%)	10 (66.7%)	13 (86.7%)	
Adjuvant therapy				0.4661
Yes	15 (50.0%)	9 (60.0%)	6 (40.0%)	
No	15 (50.0%)	6 (40%)	9 (60.0%)	
Previous radiotherapy				
Yes	7 (23.3%)	3 (20.0%)	4 (26.7%)	>0.9999
No	23 (76.7%)	12 (80.0%)	11 (73.3%)	
Recurrence				>0.9999
Yes	7 (23.3%)	4 (26.7)	3 (20.0%)	
No	23 (76.7%)	11 (73.3%)	12 (80.0%)	

Note

Data are median (IQR) or number (percentage).

### 3.5 EV‐miR‐210 expression and clinical outcome

**FIGURE 3:**
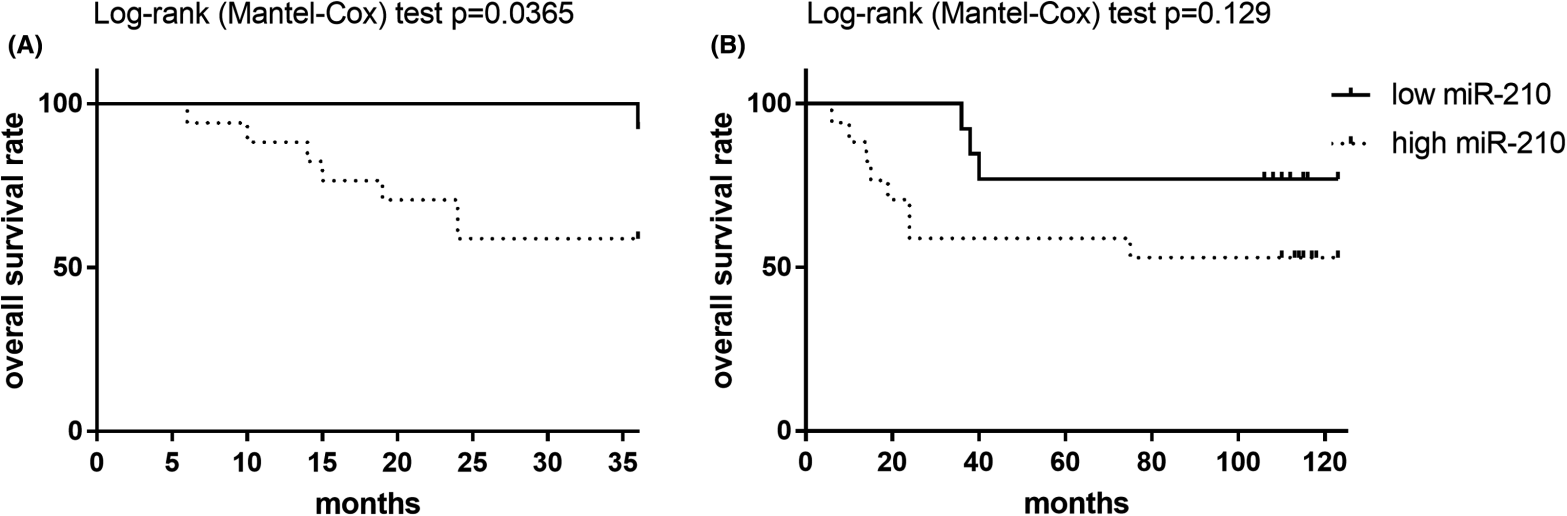
Kaplan‐Meier survival curves for patients with OSCC and high or low plasma levels of EV‐miR‐210 at three (Panel A) and ten years (end of follow‐up) after surgery (Panel B)

The median follow‐up of the study cohort was 110 months, and the overall number of deaths due to disease at 3 and 10 years were 8 (26.7%) and 11 (36.7%). Kaplan‐Meier analysis performed after dichotomization with respect to high and low EV‐miR‐210 levels showed that, 36 months after surgery, patients of the high EV‐miR‐210 group exhibited a poorer overall survival in contrast to patients with low expression levels (*p *< 0.05, Figure 3A). At the end of follow‐up (123 months after surgery), the overall survival rate of the patients tended to be comparable (Figure 3B). On multivariable Cox regression analysis with a backward selection, high EV‐miR‐210 (HR 11.69; 95% CI 1.33–102.34; *p *= 0.026), G2, and G3 grading (hazard ratio (HR) 8.13; 95% confidence intervals (CI) 1.74–37.95; *p *= 0.08), and pN >1 (HR 6.26; 95% CI 1.35–28.96; *p *= 0.019) were independent predictors of worse survival in OSCC patients.

### 3.6 **Mir‐210‐3p expression in tumor tissues from head and neck squamous cell carcinoma (HNSCC) patients according to data from The Cancer Genome Atlas**.

By using the web tool miRNA Target Viewer (miR‐TV), the ability of miR‐210a‐3p in differentiating tumor tissues from normal tissues was first analyzed using the samples from the large TCGA database. Results from 44 normal and 523 tumor tissues from HNSCC patients demonstrated that miR‐210 expression in tumors from HNSCC is significantly higher than in normal tissue (*p* = 2.2e^−16^).

## 4 DISCUSSION

High levels of miR‐210 in tumor tissues from patients with HNSCC and oropharyngeal SCC were previously reported by two independent groups^18^, ^19^ as well as in OSCC cell lines^12^ and confirmed by our analysis on miR‐TV, that provides information on miR‐210 expression in HNSCC but not, specifically, in OSCC; however, by interrogating TGCA data sets, Lu et al. (2019) showed that miR‐210‐3p was upregulated in OSCC tissues.^12^ Nevertheless, compared to tumor tissues, liquid biopsy based on body fluids offer several advantages in terms of increased accessibility, low invasiveness, low cost, and possibility of multiple sampling without discomfort.^6^ The relevance of circulating miRNAs as promising liquid biopsy tools for the early diagnosis, detection of oral cancer recurrence, and prediction of treatment response has been highlighted by recent reviews and meta‐analysis.^20^, ^21^ Despite increased serum exosomal miR‐210 expression was previously reported in glioma and renal cancer patients^13^, ^22^ raising doubts on its specificity for OSCC diagnosis, our and previous findings^12^ demonstrate that miR‐210 act as an oncomiR in OSCC and its expression is not affected by age, gender, or relevant pathological variables such as TNM classification, further reinforcing the concept that EV‐miR‐210 secretion is indeed strictly related to the presence of the OSCC *per se*. From a clinical point of view, the early detection of OSCC offers several advantages: firstly, it allows to perform a conservative organ function surgery improving the quality of life of the patient after cancer removal^23^; secondly, the early diagnosis could identify these tumors before they spread in the lymph node or invade surrounding tissue.^24^ Finally, it has been shown that the removal of OSCC in an early stage prolongs the survival rate of these patients.^25^

Some previous studies demonstrated that a number of oncogenic miRNAs are increased in EVs from OSCC patients: miR‐21 and miR‐27 were overexpressed in extracellular vesicles from OSCC patients compared to controls, but diagnostic accuracy analysis was not performed^26^; the ROC‐AUC of miR‐21 for distinguishing patients with laryngeal squamous cell carcinoma from those with vocal cord polyps was 80.1%.^27^ MiR‐512‐3p and miR‐412‐3p were also found to be upregulated in salivary extracellular vesicles from OSCC patients compared to controls with a ROC‐AUC values 0.847 and 0.871 respectively.^28^ Increased salivary EV‐miR‐24‐3p expression was found in preoperative OSCC patients compared to controls with a ROC‐AUC value of 0.738.^29^ Our ROC analysis showed that EV‐miR‐210 has an excellent diagnostic accuracy for OSCC (ROC‐AUC of 0.9513) suggesting that it may be more effective than other EV‐miRNAs in the diagnosis of OSCC.

High tissue expression of miR‐210 was previously associated with loco‐regional disease recurrence and short overall survival^18^ and correlated with tumor size and with distant metastasis^19^ in OSCC patients. Consistently, we have shown that increased levels of EV‐miR‐210 are associated with worse survival at 3 years of follow‐up in our OSCC cohort. At the end of follow‐up, the overall survival rate of patients with high or low EV‐miR‐210 was similar; however, high EV‐miR‐210 was identified as an independent prognostic factor together with well‐known adverse histological parameters such as tumor grade and pN.

The main strengths and added value of this study are (1) miR‐210 was measured in the plasma, a body fluid easily accessible and suitable for repeated sampling with minimal discomfort; (2) we demonstrated for the first time that EV‐miR‐210 is nearly absent in the plasma of healthy blood donors while it is detectable at significantly higher levels in the plasma from OSCC patients. ROC‐AUC curves analysis further indicated that EV‐miR‐210 has a great discrimination power for OSCC diagnosis and may represent a new, potential biomarker for the early detection of OSCC.

This study has also some limitations that should be acknowledged; the use of a commercial kit for EV isolation does not allow to collect an EV pellet completely free from co‐precipitated lipoproteins and vesicle‐free miRNAs. Since RNAse treatment was not performed, we cannot exclude to have measured extracellular‐free mir‐210.^30^ In fact, circulating cell‐free miRNAs can be detected in EVs or in a vesicle‐free form associated with high‐density lipoproteins or Ago2 protein and there is not a consensus on the relative abundance of miRNAs from these different sources^31^, ^32^, ^33^; however, it has been suggested that those enclosed in EVs may better reflect the presence of the tumor and therefore may serve as more specific disease biomarkers and possibly, therapeutic targets; in fact, increased abundance OSCC‐derived EVs have been reported compared to healthy controls^34^ suggesting that they may be indeed tumor‐related.^35^ The second limitation is the small number of OSCC patients. Prospective studies with larger cohorts should be performed in order to test the accuracy, and reproducibility of EV‐miR‐210 detection before it can be reliably translated into the clinical practice; moreover, looking at its potential clinical applications, future research should verify whether the levels of mir‐210 isolated from the whole plasma provide similar information. The third limitation is the unmatched control group in terms of age and smoking status, variables that do not seem to affect EV‐miR‐210 levels in OSCC patients. Lastly, EV‐miR‐210 was measured only at the time of surgery, and no additional measurement was performed in the follow‐up. We are planning to evaluate the time‐dependent variation of EV‐miR‐210 expression during the follow‐up to monitor if and when it decreases after surgical resection and if its levels raise again at tumor recurrence. This will be of particular relevance to further evaluate EV‐miR‐210 as minimal residual disease biomarker and early predictor of OSCC recurrence. Despite these limitations, the findings of this study offer new, potentially useful information for the early diagnosis OSCC and for guiding the therapeutic management of those with poor prognosis.

## 5 CONCLUSIONS

From a clinical perspective, it is critical to identify reliable and easily accessible biomarkers for non‐invasive detection of OSCC at an early stage, when conservative surgery is still practicable. Moreover, biomarkers able to predict the progression and/or recurrence of OSCC may help to reduce deaths due to this disease. Our results, despite preliminary, demonstrate that plasma EV‐miR‐210 levels hold promise for the diagnosis and prognosis of OSCC and suggest that it may also help to identify high‐risk patients, who could benefit from a more individualized treatment and follow‐up.

## CONFLICT OF INTEREST

The authors declare no potential conflict of interest.

## AUTHOR CONTRIBUTIONS

**Elisabetta Bigagli:** Conceptualization; Investigation; Methodology; Validation; Visualization; Writing – original draft; Writing – review & editing. **Luca Giovanni Locatello:** Data curation; Resources; Writing – review & editing. **Arianna Di Stadio:** Data curation; Resources; Writing – review & editing. **Giandomenico Maggiore:** Data curation; Resources; Writing – review & editing. **Francesca Valdarnini:** Formal analysis; Investigation; Writing – review & editing. **Franco Bambi:** Data curation; Resources; Writing – review & editing. **Oreste Gallo:** Conceptualization; Data curation; Resources; Writing – review & editing. **Cristina Luceri:** Conceptualization; Formal analysis; Funding acquisition; Methodology; Project administration; Software; Supervision; Validation; Visualization; Writing – review & editing.

## Supporting information

Fig S1

Table S1

## Data Availability

The data that support the findings of this study are available from the corresponding author upon reasonable request.
