# Supplementary figures and images for: Extracellular vesicles miR‐210 as a potential biomarker for diagnosis and survival prediction of oral squamous cell carcinoma patients
Source: J Oral Pathol Med. 2021 Dec 1;51(4):350–7. doi: 10.1111/jop.13263 (PMC9300091; doi:10.1111/jop.13263)

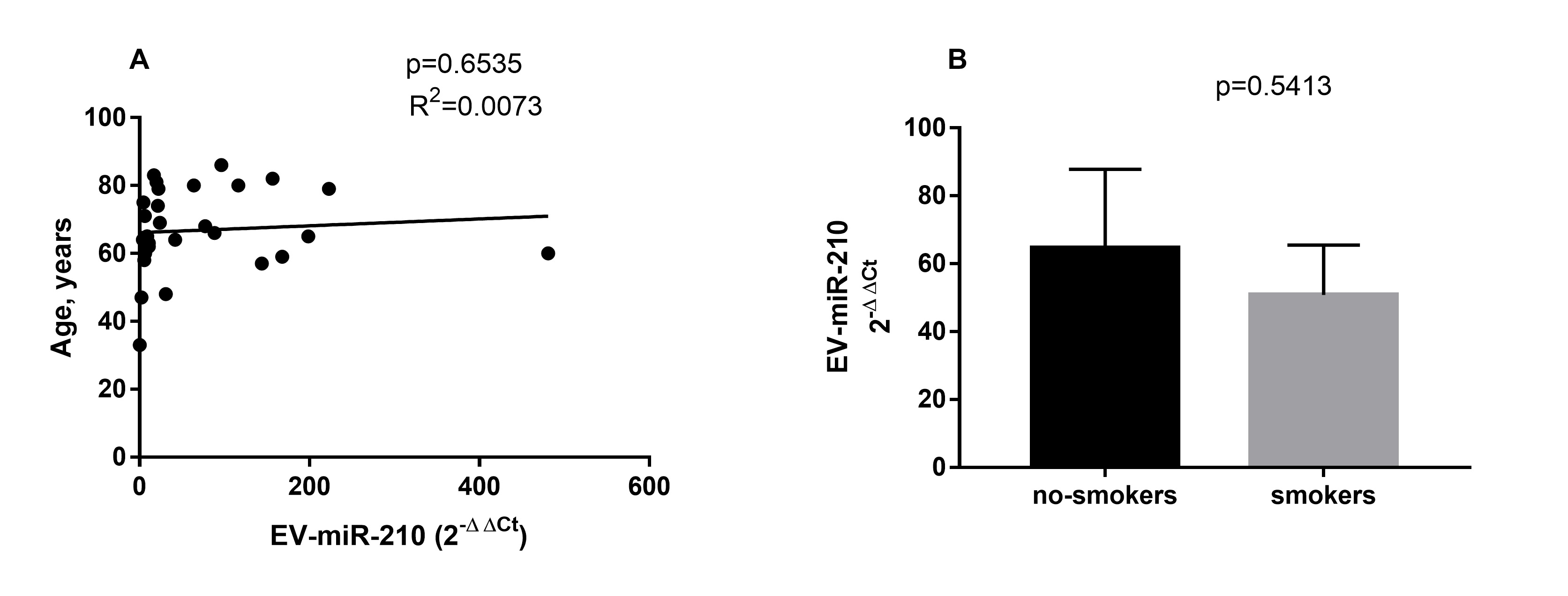

Supplement: Supplementary file 1 — Fig S1 [file JOP-51-350-s001.tif]
