# Supplementary material for: Extracellular vesicles miR‐210 as a potential biomarker for diagnosis and survival prediction of oral squamous cell carcinoma patients
Source: J Oral Pathol Med. 2021 Dec 1;51(4):350–7. doi: 10.1111/jop.13263 (PMC9300091; doi:10.1111/jop.13263)
Supplement: Supplementary file 2 — Table S1 [file JOP-51-350-s002.docx]

**Supplementary Table 1. Multiple regression analysis describing the relationship among EV-miR-210 levels and baseline characteristics of OSCC patients**

|  |  | *Standard* | *T* |  |
| --- | --- | --- | --- | --- |
| *Parameter* | *Estimate* | *Error* | *Statistic* | *P-Value* |
| CONSTANT | -78.6049 | 85.1447 | -0.923193 | 0.3715 |
| gender | 14.4148 | 35.0187 | 0.41163 | 0.6868 |
| age (years) | 0.951235 | 1.01008 | 0.941738 | 0.3623 |
| smoke habit | 58.0508 | 50.186 | 1.15671 | 0.2667 |
| alcohol use | -30.4351 | 29.7658 | -1.02249 | 0.3239 |

**Analysis of Variance**

| *Source* | *Sum of Squares* | *Df* | *Mean Square* | *F-Ratio* | *P-Value* |
| --- | --- | --- | --- | --- | --- |
| Model | 7693.22 | 4 | 1923.3 | 0.75 | 0.5716 |
| Residual | 35693.7 | 14 | 2549.55 |  |  |
| Total (Corr.) | 43387.0 | 18 |  |  |  |
